# Beyond Depression: Does the CES-D 8 Capture Subjective Well-Being?

**DOI:** 10.1177/10731911251340840

**Published:** 2025-05-24

**Authors:** Filip Fors Connolly, Tommy Gärling

**Affiliations:** 1 Umeå University, Sweden; 2 University of Gothenburg, Sweden

**Keywords:** CES-D 8, depressive symptoms, emotional well-being, measurement, subjective well-being, Sweden, validity

## Abstract

This study examines whether the 8-item Center for Epidemiologic Studies Depression Scale (CES-D 8) measures depression as distinct from the subjective well-being (SWB) components emotional well-being (EWB) and life satisfaction (LS). Using data from the Swedish European Social Survey CRONOS-2 panel (*N* = 795), we employed confirmatory factor analysis for testing discriminant validity and examined associations with health, sociodemographic, and psychological correlates. Results showed strong correlations (*r* > .70) between the latent depression factor (CES-D 8) and both latent EWB and LS factors. The depression factor furthermore failed the Fornell–Larcker tests for discriminant validity against LS and EWB, and health measures showed similar associations with CES-D 8, EWB, and LS. Limited support is thus obtained for depression being a distinct construct. CES-D 8 may primarily capture EWB rather than depression, prompting reconsideration of how depression is conceptualized and measured in relation to SWB in both research and clinical practice.

The Center for Epidemiologic Studies Depression Scale (CES-D) and its shorter counterpart CES-D 8 are among the most frequently used measures of depression at the population level (Radloff, 1977). The CES-D 8 (Karim et al., 2015) has been employed in several waves of the European Social Survey (ESS). Although both scales were intended to measure depression, recent work suggests that they may also measure a continuum spanning depression to happiness (Wood et al., 2010). This study raises the question of how much CES-D 8 overlaps with subjective well-being (SWB).

While the CES-D 8 is a shortened version of the original 20-item CES-D, it retains high internal consistency and factorial validity, making it a practical tool for large-scale surveys (Van de Velde et al., 2010). Nonetheless, depression and SWB are generally seen as distinguishable concepts. Depression is widely recognized as a health condition, especially in its clinically significant forms (American Psychiatric Association, 2013). It involves functional impairments that disrupt daily activities, work, and relationships, effects that go beyond the distress or dissatisfaction typical of low SWB. The status of depression as a health issue is reinforced by its frequent need for professional intervention, whereas low SWB, though potentially troubling, is not generally viewed as necessitating clinical treatment.

By contrast, SWB is a multifaceted construct comprising both cognitive and affective components (Diener, 1984). The cognitive component, often termed life satisfaction (LS), captures global judgments of one’s quality of life. The affective component, emotional well-being (EWB), reflects the balance between day-to-day positive and negative emotions. Although LS and EWB are positively related, with correlations typically ranging from 0.40 to 0.90 (Berlin & Fors Connolly, 2019), low scores on these measures are generally interpreted as indicators of reduced quality of life rather than as clinical health concerns.

The conceptual distinction between depression and SWB is complicated by the inherent difficulty in identifying clear boundaries across the full mood spectrum, from pathological states through normal responses to positive well-being. Nettle and Bateson (2012) note that there is no statistical point of rarity in the population distribution of depressive symptoms that offers a clear division between normal and disorder states. Any boundary between normality and disorder is inevitably somewhat arbitrary and must recognize a gradation of severity blending into normal unhappiness or low mood. This raises questions about whether mood states might best be conceptualized as existing along a single continuum from depression through normal mood to happiness, rather than as discrete categories.

Given this conceptualization of mood as potentially existing along a single continuum, the observation that the CES-D may capture a spectrum extending to happiness (Wood et al., 2010) raises important questions about what depression scales actually measure. Wood et al. (2010) demonstrated that the apparent separation between positive and negative CES-D items was methodological rather than substantive, concluding that it measures a single continuum from depression to happiness. However, they did not directly test the CES-D against established SWB scales since the analysis focused on the measure’s internal structure. Their findings nonetheless suggest that the CES-D may be capturing the same spectrum as SWB measures in an opposite way, such that high CES-D scores reflect what low SWB scores measure and low CES-D scores reflect what high SWB scores measure. If this is the case, there may be little empirical difference between depression scales like the CES-D and SWB measures, particularly with regard to the affective EWB dimension of SWB, thus further supporting the notion of a continuous spectrum from depression through normal mood to happiness.

To better understand the potential overlaps and distinctions along a depression-to-happiness continuum, it is important to examine how depression and the two components of SWB have been traditionally conceptualized. Focusing on EWB, depression and EWB differ in two key aspects. First, depression conceptualizations emphasize functional impairments, while EWB conceptualizations focus on emotional experience without addressing functional capacity. Second, depression conceptualizations traditionally place more emphasis on negative emotions than on the absence of positive emotions, while EWB is defined in a way that gives equal importance to the presence of positive emotions and the absence of negative emotions (American Psychiatric Association, 2013; Diener, 1984). Despite these different emphases in traditional conceptualizations, substantial conceptual and empirical overlap exists between depression and EWB, as both constructs address negative emotions, and several depression conceptualizations explicitly include low positive affect as an important feature (Clark & Watson, 1991; Fried et al., 2022). This overlap supports the possibility of a unified continuum model.

Turning to LS, depression primarily concerns emotional states and associated functional impairments, whereas LS represents a cognitive evaluation of one’s life circumstances against self-selected standards (Diener, 1984). In principle, one could experience depression yet still judge life circumstances in a relatively positive way, or be dissatisfied with life without being depressed. Nevertheless, because depression often involves pervasive negative biases in memory and judgment (LeMoult & Gotlib, 2019), one would still anticipate considerable empirical overlap between depression and LS, even if remaining theoretically distinct.

The theoretical similarities and differences between depression and SWB raise important questions about what population-level depression screening tools like the CES-D 8 actually measure. Understanding the empirical relationship between CES-D 8 and established SWB measures has important implications for both research and clinical practice. If substantial overlap is found, this would suggest that large population surveys using CES-D 8 and similar depression measures could provide valuable insights into SWB patterns at the population level. Moreover, strong empirical connections between these constructs would indicate potential benefits from integrating research traditions. Insights about the determinants and consequences of SWB could inform the understanding of depression risk factors and outcomes, while depression research could enhance knowledge of SWB. However, if CES-D 8 shows different patterns from SWB measures, this would suggest that depression measurements of this style capture important differences between depression and variations in SWB.

## This Study

Despite the potential implications, a comprehensive analysis of the relationship between CES-D and SWB is lacking. While some studies have suggested that CES-D may measure SWB (Wood et al., 2010), the extent to which CES-D captures the cognitive and affective components of SWB remains unclear due to a lack of proper analysis of discriminant validity. Furthermore, previous research has not adequately examined whether CES-D and SWB have similar or distinct correlates. This study aims at addressing these knowledge gaps by investigating the overlap between CES-D 8 and SWB using data from the ESS CRONOS-2 panel in Sweden, a context in which SWB levels are comparatively high by international standards (Helliwell et al., 2023) at the same time as depression and related mental health issues remain significant (Höglund et al., 2020). Although the research question is not country specific, Sweden thus provides an appropriate test case for examining whether CES-D 8 distinguishes depression from SWB.

Specifically, the aim of this study is (a) to assess the discriminant validity of CES-D 8 and established measures of LS and EWB using confirmatory factor analysis (CFA), and (b) to examine the relationships of key health (self-reported functional ability and general health), sociodemographic (age, gender, and income), and psychological correlates (personality traits and need fulfillment) with latent factors of depression (CES-D 8), EWB, and LS.

We hypothesize that if the CES-D 8 captures depression as a psychological construct distinct from SWB, this should be evident in two ways. First, we will employ CFA to examine the factor loadings, that is, how strongly each item relates to its proposed construct, and assess the discriminant validity between constructs by comparing the average variance extracted (AVE) values to the squared correlations between factors (Fornell & Larcker, 1981; Hair et al., 2009). If the indicators of the CES-D 8 reflect a coherent and unique depression construct, rather than simply reflecting LS or EWB, we expect to observe strong factor loadings on their respective factors and AVE values higher than the squared correlations between factors. This statistical approach allows us to determine whether the CES-D 8 measures a construct that is distinct from the SWB components or simply captures the opposite of the SWB spectrum.

Second, we posit that if the CES-D 8 measures depression as a distinct construct, it should exhibit different patterns of relationships with key correlates compared to LS and EWB and also demonstrate incremental explanatory variance beyond these measures. This is particularly relevant for health indicators, as depression is considered a health problem, whereas low LS and low EWB are not typically considered as health issues. Specifically, we expect that depression, as measured by the CES-D 8, will show stronger associations with self-reported functional ability and general health evaluations than LS and EWB. Furthermore, if the CES-D 8 captures a construct distinct from LS and EWB, it should be expected to display some degree of differentiation in relation to key sociodemographic and psychological factors. We test the differential relationships with age, gender, income, personality traits, and psychological needs, which all may have distinct patterns of association with clinically relevant health problems (like depression) compared to broader SWB constructs. While we do not advance specific hypotheses about the precise nature of the differentiation for each variable, the potential for functional limitations associated with depression but not SWB provides a basis for expecting overall differences. By examining both the patterns of correlations and the incremental explained variance of the measures in relation to these factors, we can assess whether the CES-D 8 captures unique aspects of psychological functioning beyond low SWB. This analysis complements the CFA by providing evidence from both correlational patterns and incremental variance analyses that the CES-D 8 measures something distinct from SWB.

## Method

### Participants and Procedure

To investigate the research questions, we used panel data from the ESS collected in Sweden. We focused on Swedish data because waves 3 and 6 of the CRONOS-2 panel employed country-specific questionnaires, and the key measures of SWB were not available for the other countries participating in CRONOS-2.

**Figure 1.**
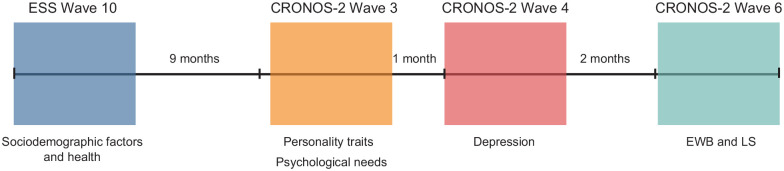
Data collection timeline for ESS wave 10 and CRONOS-2 waves 3, 4, and 6 in Sweden. *Note.* Data collected exclusively in Sweden due to country-specific questionnaires in CRONOS-2 Rounds 3 and 6. ESS = European Social Survey; EWB = emotional well-being; LS = life satisfaction.

The ESS is based on samples representative of all individuals aged 15 years and older, with random samples drawn from each of the eight NUTS-2 (Nomenclature of Territorial Units for Statistics, level 2) regions to construct the national sample. Data from multiple sources were merged. First, the Swedish ESS wave 10 (*n* = 2,308; response rate 37.9%) was collected via paper and web surveys from October 10, 2021, to January 17, 2022, providing measures of sociodemographic factors and health. A subset of these participants (*n* = 1,443) was recruited to the ESS CRONOS-2 web panel, allowing linkage to additional data. For this study, we used CRONOS-2 wave 3 (August 31 to September 29, 2022; *n* = 849; response rate 59.2%), wave 4 (October 10 to November 9, 2022; *n* = 845; response rate 57.8%), and wave 6 (January 9 to February 8, 2023; *n* = 811; response rate 56%), which all include the measures of CES-D 8, LS, EWB, personality traits, and psychological need fulfillment. The timing of the waves is shown in Figure 1.

All datasets were collected in compliance with international ethical standards, including GDPR regulations and the International Statistical Institute’s ethical declaration, under oversight from the ESS ERIC Research Ethics Board/Committee. All participants provided informed consent before participation in accordance with the ESS Privacy and Data Protection policy, and no personally identifying information was accessible to the researchers.

### Measures

#### Life Satisfaction

Items tapping the cognitive and emotional components of SWB were included in CRONOS-2 wave 6. LS was assessed using the standard ESS measure: “All things considered, how satisfied are you with your life as a whole nowadays?” with end points labeled “0 Extremely dissatisfied” and “10 Extremely satisfied.” Though this is a single-item measure, it has been demonstrated to have satisfactory convergent validity with multi-item LS scales (Fors & Kulin, 2016). In addition, four items taken from the Riverside Life Satisfaction Scale (Margolis et al., 2019) were used. Participants indicated their level of agreement with two positive statements and two negative statements using a seven-point verbal scale, where 1 represents “Strongly Disagree” and 7 represents “Strongly Agree.” The statements were: “I am content with my life,” “Those around me seem to be living better lives than my own,” “I am satisfied with where I am in life right now,” and “I want to change the path my life is on.” Previous research has demonstrated strong psychometric properties for these items, with factor loadings ranging from 0.92 to 0.96 for the positive items and −0.66 to −0.83 for the negative items, as well as convergent validity (Margolis et al., 2019).

#### Emotional Well-being

EWB was assessed by means of retrospective ratings of the frequency of emotions experienced during the past week. Six unipolar adjective scales with seven steps ranging from “never” (0) to “always” (6) were used. Each scale is defined by three adjectives taken from the Swedish Core Affect Scale (SCAS) (Västfjäll & Gärling, 2007). Participants indicated on three of the scales how often during the past week they felt positive emotions high in activation (engaged, interested, optimistic), neutral in activation (glad, happy, pleased), and low in activation (serene, calm, relaxed). On the other three scales, participants indicated how often they felt negative emotions high in activation (tense, anxious, nervous), neutral in activation (sad, depressed, displeased), and low in activation (bored, indifferent, pessimistic). The selected SCAS items has been shown in Swedish samples to have Cronbach’s alpha coefficients ranging from .78 to .85 (Fors Connolly & Gärling, 2024; Gärling et al, 2016).

#### Depression

Depression was assessed using the CES-D 8, a widely validated 8-item short form of the original 20-item CES-D Scale (Karim et al., 2015). Participants reported the frequency of experiencing various feelings and behaviors during the past week, including both negative symptoms (e.g., “I felt depressed”) and positive emotions (e.g., “I was happy”), as well as items exploring sleep disturbances, lack of motivation, and feelings of loneliness. Each item was rated on a four-point frequency scale ranging from 1 (“Rarely or none of the time”) to 4 (“Most or all of the time”), with higher scores indicating more depressive symptoms. Multiple studies have supported the factorial validity and internal consistency of the CES-D 8 in diverse populations (Van de Velde et al., 2010). Cronbach’s alpha coefficients have generally ranged from .73 to .88.

While in the official English translations of the CES-D 8 overlaps with SCAS by using “happy” and “depressed,” the Swedish versions in this study used different adjectives. For “happy,” CES-D 8 in the ESS uses “lycklig” while SCAS uses “munter.” Similarly, for “depressed,” CES-D 8 uses “deprimerad,” while SCAS uses “nedslagen.” The only adjective that is identical across both scales in Swedish is “sad” (“ledsen”).

#### Health Status

Two items assessed participants’ health status. The first item measured activity limitation due to health conditions (“Are you hampered in your daily activities in any way by any longstanding illness, or disability, infirmity or mental health problem?”) with three response options (1 *Yes*, a lot; 2 *Yes*, to some extent; 3 *No*). Self-rated general health was measured with a single item (“How is your health in general?”) rated on a 5-point scale (1 *Very good*; 2 *Good*; 3 *Fair*; 4 *Bad*; 5; *Very bad*). These single-item health indicators are standard measures widely used in population health surveys and have shown predictive validity for health outcomes in previous research (Idler & Benyamini, 1997).

#### Personality

To measure the Big Five personality traits, the 15-item BFI-2-XS Scale (Rammstedt et al., 2018) was employed. In a validation study, Rammstedt et al. (2018) demonstrated acceptable psychometric properties for this extra-short version, with test–retest reliabilities ranging from 0.66 to 0.84 and Cronbach’s alpha coefficients ranging from .45 to .73 across the five traits. For each of the five traits, three-item indexes were constructed: Extraversion (α = .54), Agreeableness (α = .52), Conscientiousness (α = .54), Neuroticism (α = .74), and Openness to Experience (α = .42).

#### Psychological Needs

Need satisfaction in terms of Competence (α = .72), Relatedness (α = .74), and Autonomy (α = .78) was assessed using three-item indexes for each construct. These items were adapted from previous studies (Chen et al., 2015; Sheldon et al., 2001).

#### Socioeconomic Factors

Household income was assessed using participants’ reports of their total annual household net income, based on the Swedish income distribution categorized into 10 income brackets (deciles) presented in Swedish kronor (SEK). Gender was recorded as male or female. Age was calculated based on participants’ reported year of birth.

### Data Analyses

All data analyses were conducted using R version 4.3.1 (R Core Team, 2023) with the lavaan package (Rosseel, 2012) for the CFA analyses. The analysis of the factor structure and construct validity was conducted using full information maximum likelihood (FIML) estimation to handle the missing data. FIML was chosen as it provides unbiased parameter estimates given data are missing at random. In the correlational analysis, we computed Pearson correlations to examine the relationships between each predictor variable and latent measures of LS, EWB, and depression (inverted to align its directionality with the well-being measures). For these analyses, we included only participants with complete data for all variables (*n* = 631) to ensure consistency across comparative analyses requiring paired observations. To compare the relative strength of correlations, we employed Williams’ test for dependent correlations using the *r*.test function from the psych package in R. Using the same sample, we then assessed incremental variance explained through a series of hierarchical regression analyses. While often considered predictors of well-being, the key correlates were treated as dependent variables in these specific analyses. This approach allowed us to quantify the unique portion of variance in these correlates explained by depression when controlling for SWB (LS or EWB), and conversely, the variance explained by SWB (LS or EWB) when controlling for depression. For each key correlate, we conducted four analyses to examine: (a) the additional variance explained by depression over LS, (b) the additional variance explained by depression over EWB, (c) the additional variance explained by LS over depression, and (d) the additional variance explained by EWB over depression. The change in *R*-squared (Δ*R*^2^) between these models indicates the unique contribution of each measure relative to the other in these specific comparisons. It is important to note that this analytical approach, treating correlates as outcomes, serves primarily to isolate unique statistical associations; it does not imply a theoretical causal direction from SWB/depression to these correlates, nor should these associative findings be interpreted causally based on the correlational analyses performed.

## Results

### Factor Structure and Construct Validity

From the total sample of 863 participants, 795 cases were included in the CFA, with 26 different missing data patterns identified. The comparative fit index of 0.92 and Tucker–Lewis index of 0.91 are close to the recommended threshold of 0.95. The root mean square error of approximation of 0.08 (90% CI [0.07, 0.08]) indicates an acceptable fit, while the standardized root mean square residual of 0.05 suggests a good fit (Hu & Bentler, 1999).

Examining the factor loadings (see Table 1), we observe that all items load significantly on their respective factors. For the LS factor, standardized loadings range from 0.62 to 0.90. The EWB factor shows loadings between 0.71 and 0.80, while the depression factor has loadings from 0.57 to 0.84. The loadings indicate convergent validity within each construct but with higher validity for LS and EWB compared to depression. LS and EWB show a positive correlation (*r* = .82), which is expected given that they are both components of SWB and measured at the same survey occasion. The latent depression factor extracted from the CES-D 8 has negative correlations with both LS (*r* = −.70) and EWB (*r* = −.75), suggesting substantial overlap between these constructs, despite being measured during different occasions.

**Table 1:** Standardized Factor Loadings for Indicators of Life Satisfaction, Emotional Well-Being, and Depression (CES-D 8), Standard Errors, and Lower and Upper 95% Confidence Intervals.

Latent factor	Indicator	Loading	Standard error	*z*-Value	Lower 95% CI	Upper 95% CI
LS	LS1	0.90	0.01	91.38	0.88	0.92
	LS2	0.86	0.01	75.31	0.84	0.89
	LS3	−0.62	0.02	−25.79	−0.67	−0.58
	LS4	0.85	0.01	67.57	0.82	0.87
	LS5	−0.63	0.02	−26.30	−0.68	−0.59
EWB	Glad, happy, pleased	0.78	0.02	46.77	0.75	0.82
	Serene, calm, relaxed	0.79	0.02	49.952	0.76	0.83
	Engaged, interested, optimistic	0.75	0.02	39.988	0.71	0.79
	Sad, depressed, displeased	−0.80	0.02	−49.921	−0.83	−0.77
	Tense, anxious, nervous	−0.75	0.02	−40.014	−0.79	−0.71
	Bored, indifferent, pessimistic	−0.71	0.02	−34.655	−0.75	−0.67
Depression (CES-D 8)	Depressed	0.84	0.01	59.428	0.81	0.87
	Effort	0.69	0.02	31.781	0.65	0.73
	Restless	0.60	0.03	23.280	0.55	0.65
	Happy	−0.58	0.03	−22.169	−0.64	−0.53
	Lonely	0.66	0.02	28.274	0.61	0.70
	Enjoyed life	−0.57	0.03	−20.997	−0.62	−0.52
	Sad	0.80	0.02	50.796	0.77	0.83
	Not going	0.59	0.03	21.813	0.53	0.63

*Note*. Values below the lower CI and above the upper CI are considered statistically significant at the 0.05 level. The model included an estimated residual correlation between the “Happy” and “Enjoy” indicators within the Depression (CES-D 8) factor due to shared method variance associated with positively worded items. LS = life satisfaction; EWB = emotional well-being; CES-D 8 = 8-item Center for Epidemiologic Studies Depression Scale; CI = confidence interval.

To assess discriminant validity, specifically addressing whether the CES-D 8 (Depression) measure captures a construct distinct from the LS and EWB, we applied the Fornell and Larcker (1981) criterion comparing AVE against squared inter-factor correlations. The analysis yielded AVE values of 0.61 for LS, 0.58 for EWB, and 0.45 for depression, with squared correlations of .66 (LS-EWB), .50 (LS-Depression), and .57 (EWB-Depression). The results first indicate a lack of discriminant validity between the two SWB components, LS and EWB, as neither factor’s AVE exceeds their high squared correlation (.66). Regarding the primary question of depression’s distinctiveness from SWB, the Fornell–Larcker criterion is not met when evaluating the depression factor itself; its AVE (0.45) is lower than its squared correlations with both LS (0.50) and EWB (0.57). Although both LS and EWB do meet the criterion relative to depression based on their own AVEs (with LS showing a notably larger margin of distinction than EWB), the failure of the depression factor’s AVE to exceed the shared variance with the SWB components suggests that, according to this specific metric, the CES-D 8 does not, in this analysis, demonstrate sufficient discriminant validity to be considered distinct from LS and EWB.

### Criterion-related Validity: Differential Associations and Incremental Explanatory Variance

**Figure 2.**
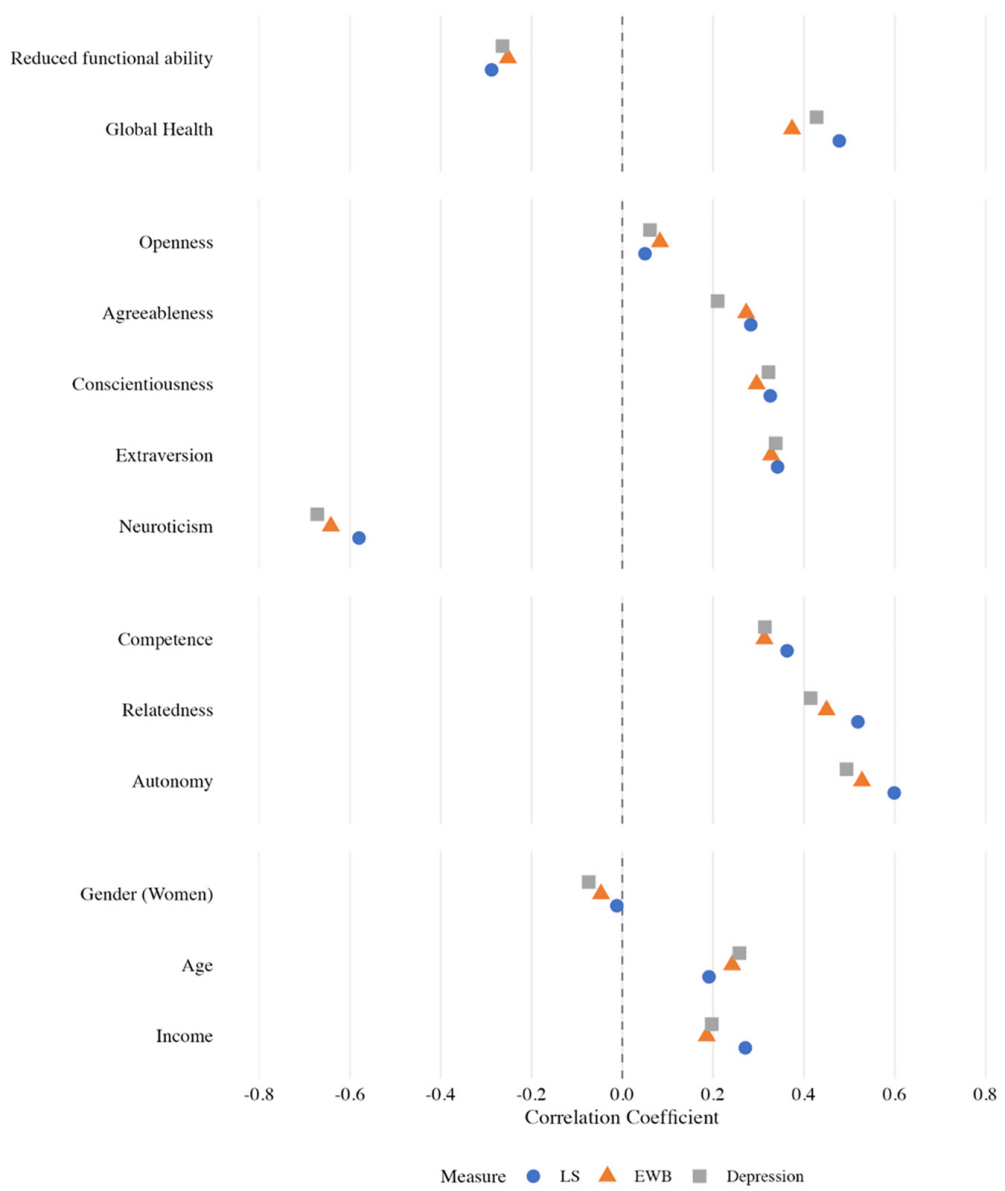
Pearson correlations between latent factors of LS, EWB, and depression (CES D-8) with health, personality traits, psychological needs, and sociodemographic variables. *Note.* LS = life satisfaction; EWB = emotional well-being; CES-D 8 = 8-item Center for Epidemiologic Studies Depression Scale.

**Figure 3.**
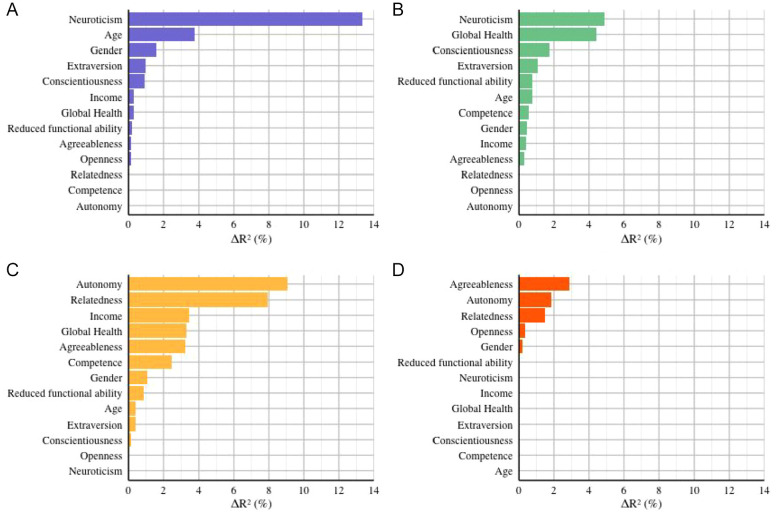
Incremental validity analyses of latent factors of depression (CES D-8, life satisfaction, and emotional well-being. (A) Depression over life satisfaction. (B) Depression over emotional well-being. (C) Life satisfaction over depression. (D) Emotional well-being over depression. *Note.* CES-D 8 = 8-item Center for Epidemiologic Studies Depression Scale.

Our analyses examined the differential associations of the latent factors of CES-D 8 (hence referred to as depression), LS, and EWB with key correlates as well as their unique contributions to explaining the variance in the correlates treated as outcomes. For ease of interpretation, we reverse-scored depression so that higher scores reflect lower depressive symptoms. Results of the bivariate Pearson correlations are displayed in Figure 2 and Appendix Table A1, while the incremental variance analyses are presented in Figure 3 (see Appendix Table A2 for detailed results).

The health measures should be more strongly related to depression than LS and EWB, but the analysis does not confirm this. Instead, global health showed the strongest correlation with LS (*r* = .48), followed by depression (*r* = .43), and the weakest correlation with EWB (*r* = .37). These differences were all statistically significant (*p*s < .029), suggesting that perceived health status may be more integral to judgments of LS than to either EWB or depression. The pattern for reduced functional ability was more uniform, demonstrating modest negative correlations of similar magnitude across all three measures (*r*s = −.25 to −.29) with no significant differences between them. This consistent pattern suggests that functional limitations may impact all aspects of well-being similarly, rather than having a specific stronger relationship with depression as might be expected.

Of the personality traits, Neuroticism emerged as the strongest correlate, showing substantial negative correlations (LS: *r* = −.58, EWB: *r* = −64, depression: *r* = −.67), with significant differences between LS and both the other measures (*p*s < .001). Extraversion showed consistent positive correlations (*r*s = .33 to .34) with no significant differences. Agreeableness had positive correlations that were significantly stronger for LS (*r* = .28) and EWB (*r* = .27) than depression (*r* = .21; *p*s < .006). Conscientiousness showed moderate positive correlations (*r*s = .30 to .33), while openness had weak correlations (*r*s = .05 to .08), neither showing significant differences between measures.

Psychological needs had consistently positive associations. Autonomy showed the strongest correlations (LS: *r* = .60, EWB: *r* = .53, depression: *r* = .49) with significant differences between LS and both other measures (*p*s < .001). Similarly, relatedness had positive correlations (LS: *r* = .52, EWB: *r* = .45, depression: *r* = .42) with significant differences between LS and both the other measures (*p*s < .001). Competence correlations (*r*s = .31 to .36) also showed significant differences between LS and both the other measures (*p*s < .039).

The results for sociodemographic variables were varied. Age had positive correlations across measures (LS: *r* = .19, EWB: *r* = .24, depression: *r* = .26), with significant differences between LS and both other measures (*p*s < .010). Gender had negative correlations (*r*s = −.01 to −.07), with the only significant difference being between LS and depression (*p* = .015), indicating that women scored slightly higher on depression but did not differ from men on LS. Income had stronger correlations with LS (*r* = .27) than EWB (*r* = .19) and depression (*r* = .20), with significant differences between LS and both other measures (*p*s < .003).

Overall, of the 13 predictors examined, 8 showed significant differences between LS and depression, 4 between EWB and depression, and 7 between LS and EWB. This pattern suggests a higher degree of similarity between depression and EWB in their associations with key correlates, while LS demonstrates greater distinctiveness.

While correlation patterns provide important insights into the relationships between well-being measures and key correlates, they do not quantify the unique portion of variance in the correlates that each well-being measure explains when controlling for the others. To address this, we conducted hierarchical regression analyses examining the additional variance explained by each measure. As shown in Figure 3 and Appendix Table A2, adding depression to LS led to increases in explained variance (Δ*R*^2^) across several correlates, particularly for neuroticism (13.35%) relative to LS alone. Depression added some variance for age (3.74%) and gender (1.57%). Adding depression to EWB also improved several predictions, with contributions for neuroticism (4.91%), global health (4.40%), and smaller contributions for conscientiousness (1.77%) and extraversion (1.07%). In contrast, LS contributed beyond depression for some correlates, particularly for autonomy (9.09% additional explained variance), relatedness (7.91%), and competence (2.47%). This indicates that LS has relationships with these correlates that are not accounted for by depression. Similarly, LS added some explanatory variance for agreeableness (3.25%), global health (3.29%), and income (3.43%). EWB also had positive incremental effects, particularly for agreeableness (2.88%), autonomy (1.84%), and relatedness (1.47%). The strongest associations independent of depression consistently emerged for neuroticism, while LS showed its largest unique variance explained in relation to the psychological need measures. However, these comparisons should be interpreted with caution. Because depression and the SWB measures (LS and EWB) share considerable empirical overlap, estimates of the unique variance explained by each may be less precise, particularly for the smaller effects. Additionally, the multiple comparisons increase the likelihood of significant differences due to chance alone.

## Discussion

### Summary of Results

Although the CES-D 8 was designed to screen for depressive symptoms, our analysis reveals substantial overlap with measures of SWB, particularly with its affective component. Specifically, the CFA indicated that the latent depression factor (depression), derived from the CES-D 8 items, lacks discriminant validity against both EWB and LS. The strong negative correlations between depression and both LS (*r* = −.70) and EWB (*r* = −.75) suggest substantial overlap among these constructs. Notably, the strong correlations persist even though the survey items for depression and for LS and EWB were administered about 2 months apart. To formally assess discriminant validity, we compared the AVE for each factor with the squared correlations between factors based on the Fornell–Larcker criterion (Fornell & Larcker, 1981). The results indicated a lack of discriminant validity between LS and EWB. While LS showed clear distinctiveness from depression, and EWB showed borderline distinctiveness from depression based on their respective AVEs exceeding the shared variance, but crucially the depression factor itself failed to meet the discriminant validity criterion against either of the two SWB components. Our findings contradict the assumption that CES-D 8 constitutes its own depression construct separate from SWB.

In the second part of the analysis, we examined the relationships between correlates of depression, EWB, and LS, as well as their incremental explained variance. The results further highlighted the similarity between depression and EWB and to a lesser extent LS. Demographic factors (age, gender, income) and personality traits (extraversion, agreeableness, neuroticism) showed similar patterns for depression and EWB, whereas LS displayed a more distinct pattern. Psychological needs (autonomy, relatedness) showed strong positive relationships across all outcomes, but with stronger associations with LS. Incremental variance analyses indicated that depression consistently added unique variance to neuroticism, whereas LS contributed more to need satisfaction. These findings underscore the close relationship between CES-D and EWB, while distinguishing both from LS. Notably, the two health measures, self-reported functional ability and general health, were no more strongly related to depression than LS, and only one showed a marginally stronger link with EWB. This is noteworthy given that depression is usually conceptualized as a health problem, whereas low LS and EWB are not.

### Methodological Issues

We used the SCAS as the EWB measure. While the SCAS is less commonly adopted in SWB research compared to scales like the positive and negative affect schedule (PANAS) and the scale of positive and negative experience (SPANE) (Jovanović et al., 2019), it has distinctive features that are relevant to our findings. Notably, the SCAS is based on the circumplex model of affect, which conceptualizes emotions along the two primary dimensions valence (ranging from positive to negative) and arousal or activation (ranging from high to low) (Russell, 1980). In this model, positive and negative emotions are positioned at opposite ends of a bipolar dimension of affective experience. In contrast, alternative EWB measures like the PANAS conceptualize positive and negative affects as independent dimensions (Watson et al., 1988). The PANAS has faced criticism as an EWB measure because it predominantly focuses on high-arousal states like stress and excitement, lacking items targeting prototypical positive and negative emotions with neutral arousal, such as happiness and sadness, or low-arousal emotions such as calmness and boredom. However, the SPANE (Jovanović et al., 2019), which is similar to the SCAS in capturing a wide range of affective experiences with varying levels of arousal, might also exhibit overlap with depression measures. This raises the methodological issue that the theoretical framework and item content of EWB scales may influence their overlap with depression measures like the CES-D 8. Using scales based on independent affect dimensions, like the PANAS, might reduce this overlap. However, the PANASs limited coverage of the full affect spectrum challenges its suitability as a comprehensive EWB measure.

The assessment of construct validity in survey research is often carried out with measurements collected during the same survey occasion. This may artificially reduce discriminant validity between constructs due to current mood bias effects (Berlin & Fors Connolly, 2019). However, since the measures of depression and SWB were collected during different occasions, we should be able to rule out the effects of current mood explaining the main results. On the other hand, the overlap between LS and EWB might have been stronger due to this bias, as they were measured on the same occasion.

While a majority of SWB research has historically centered on LS, there has been a recent interest in EWB. For instance, a study showing that income is more strongly related to LS than to EWB has been cited more than 3,000 times since 2010 (Kahneman & Deaton, 2010). Despite this, numerous population surveys fall short of including proper EWB measures. Instead, they often feature single-item questions about happiness, like “How happy are you?” which some researchers interpret as indicative of EWB. While such questions are not inherently flawed, their reference to one’s overall life, rather than a specific time frame, positions them as hybrid indicators of LS and EWB. Given these considerations, and the fact that a multi-item scale usually has higher reliability, we recommend adopting the CES-D 8, or a selection of its items, as a more suitable measure of EWB for researchers working with surveys that encompass both measures.

It is worth noting that while this study focused on the CES-D 8, the original 20-item CES-D might offer a more nuanced assessment of depression. The full CES-D includes additional items covering specific symptoms and experiences such as appetite changes, social interactions, and cognitive symptoms, which are markers for functional ability. While the CES-D 8 retains some functional ability indicators, it contains fewer of these markers compared to the full scale. This broader coverage might potentially reduce the overlap with SWB measures. However, the strong overlap we observed between the CES-D 8 and EWB suggests that even the more comprehensive CES-D may still face challenges in discriminating between depression and EWB. This possibility is further strengthened by the fact that both the CES-D 8 and the full CES-D maintain a similar ratio of positively worded items (2 out of 8 in CES-D 8, and 4 out of 20 in the full CES-D).

Another important methodological issue concerns item overlaps between the CES-D 8 and EWB as measured by the SCAS. While overlap exists, particularly in items capturing negative affect like sadness, we argue that this overlap is theoretically necessary. Certain experiences, such as sadness, are inherently central to both depression and low EWB because they are defining features that cannot be excluded without compromising content validity. In depression measures, sadness is viewed as a core symptom, often accompanied by less central symptoms such as sleep problems and concentration difficulties. In EWB measures, sadness is often viewed as a prototypical negative emotion. The critical question is, therefore, whether these constructs, despite sharing core indicators, demonstrate sufficiently distinct patterns of psychometric properties to justify treating them as separate constructs.

This study was conducted using only a Swedish sample, and consequently, the CES-D 8 was administered in Swedish. While there is no obvious reason to expect that the findings would not generalize to other countries and languages, it is important that future research replicate this study in different cultural contexts. Such replication would help confirm the generalizability of the results and ensure that the observed overlaps between depression measures and SWB are consistent across diverse populations.

### Theoretical and Practical Implications

The findings raise the question of whether the CES-D 8 is more adequately conceptualized as a measure of EWB than depression. While depression is traditionally viewed as a mood disorder characterized by persistent negative feelings (Beck et al., 1961), the absence of positive emotions is also recognized as a significant component (Clark & Watson, 1991). This aligns with broader concerns in depression measurement. Fried et al. (2022) highlighted challenges in content validity and dimensionality, noting a lack of consensus across depression scales as well as their multidimensional nature. It is furthermore unclear whether depression is most appropriately viewed as a latent factor or a cluster of distinct symptoms that commonly co-occur (e.g., Beard et al., 2016). These issues may stem from the lack of explicit theories about the nature of depression. Without clear theoretical foundations, it remains uncertain what the scales are aimed to measure and how to evaluate their effectiveness. It is worth noting that SWB also remains a contested construct in both conceptualization and measurement (e.g., Kashdan et al., 2008). However, the central distinction between LS and EWB, and how these components are defined and measured, still appears theoretically clearer and more distinct compared to depression. These conceptual challenges may have practical implications for both research and clinical applications.

Our findings reveal a considerable overlap between depression, as measured by the CES-D 8, and SWB components, particularly EWB. This challenges the traditional view of these measures assessing distinct constructs, at least within population screening contexts. The generality of this conclusion, however, depends on how depression is defined and the extent to which the CES-D 8 represents depression screening tools more broadly. Specifically, the very large overlap with EWB suggests that the CES-D 8 might function more effectively as a proxy for EWB in surveys lacking dedicated measures for it, while the more moderate association with LS confirms that cognitive judgments of LS retain a larger degree of distinctiveness. These results highlight the potential value in further integrating the research traditions of SWB and depression. Several avenues for future research emerge from these findings. Further investigation is needed to explore the theoretical boundaries between depression and low EWB more rigorously and to evaluate whether our findings generalize to other commonly used depression measures. Relatedly, researchers should consider if screening tools like the CES-D 8 are perhaps more accurately conceptualized as measures of (low) EWB rather than depression per se, as our latent factor analysis indicates. Furthermore, the clinical implications warrant examination, including whether the operationalization of depression in some settings needs reconsideration and whether interventions targeting EWB could be beneficial for depression prevention and treatment.

## Conclusion

In conclusion, this study reveals a substantial overlap between the CES-D 8 and a measure of EWB, challenging traditional conceptualizations of tools for depression screening. This overlap may partly stem from the unclear nature of depression itself, with ongoing debates about its definition, key symptoms, and measurement. The lack of discriminant validity and similar patterns of associations with key correlates suggest that the measures may be tapping into a common construct. This finding has significant implications for mental health research and practice, as well as highlighting the potential utility of the CES-D 8 as an EWB measure in large-scale surveys. This study contributes to the ongoing dialogue about the nature of depression and well-being, calling for a more integrated approach to their conceptualization and measurement.

### Use of Large Language Models

In the manuscript preparation phase, we employed Claude 3.5 Sonnet, a Large Language Model (LLM) developed by Anthropic, to assist with language checks, proofreading, and ensuring clarity of expression. All suggestions provided by the LLM were carefully reviewed and selectively implemented by the authors to maintain the integrity and accuracy of the scientific content.

## Appendix

**Table A1:** Pearson Correlations of Latent Factors Corresponding to Life Satisfaction, Emotional Well-Being, and Depression (CES-D 8) with Health, Personality, Psychological Needs, and Sociodemographic Variables.

Predictor	Pearson correlations			Comparisons (*p*-values)		
	LS	EWB	Depression	LS vs. EWB	LS vs. depression	EWB vs. depression
Health						
Global health	.48	.37	.43	<.001	.029	.013
Reduced functional ability	−.29	−.25	−.26	.065	.339	.591
Personality traits						
Extraversion	.34	.32	.34	.470	.860	.671
Agreeableness	.28	.27	.21	.605	.003	.006
Conscientiousness	.33	.30	.32	.111	.855	.246
Neuroticism	−.58	−.64	−.67	<.001	<.001	.084
Openness	.05	.08	.06	.106	.688	.347
Psychological needs						
Competence	.36	.31	.31	.007	.039	.960
Autonomy	.60	.53	.45	<.001	<.001	.097
Relatedness	.52	.45	.42	<.001	<.001	.095
Demographic variables						
Age	.19	.24	.26	.010	.007	.475
Gender (women)	−.01	−.05	−.07	.086	.015	.247
Income	.27	.19	.20	<.001	.003	.653

*Note.* CES-D 8 = 8-item Center for Epidemiologic Studies Depression Scale; EWB = emotional well-being; LS = life satisfaction.

**Table A2:** Hierarchical Regression Analyses with Well-Being Correlates Treated as Dependent Variables and Latent Factors Corresponding to Life Satisfaction, Emotional Well-Being, and Depression (CES-D 8) as Independent Variables.

	Analysis 1					
	Step 1: LS		Step 2: LS+depression			
Well-being correlates	β_LS_	*R* ^2^	β_LS_	β_Depression_	*R* ^2^	Δ*R*^2^
Extraversion	.36***	.13	.21**	.19**	.14	.01
Agreeableness	.29***	.09	.32***	−.04	.09	.00
Conscientiousness	.34***	.12	.19**	.19**	.13	.01
Neuroticism	−.61***	.37	−.13*	−.61***	.50	.13
Openness	.05	.00	.00	.06	.00	.00
Competence	.38***	.14	.32***	.08	.14	.00
Autonomy	.62***	.39	.57***	.06	.38	.00
Relatedness	.54***	.29	.53***	.02	.29	.00
Global health	.50***	.25	.39***	.14*	.25	.00
Reduced functional ability	−.30***	.09	−.22***	−.11	.09	.00
Age	.20***	.04	−.04	.31***	.08	.04
Gender (women)	−.01	.00	.13	−.18**	.02	.02
Income	.28***	.08	.32***	−.05	.08	.00
	Analysis 2					
	Step 1: EWB		Step 2: EWB+depression			
Well-being correlates	β_LS_	*R* ^2^	β_EWB_	β_Depression_	*R* ^2^	Δ*R*^2^
Extraversion	.35***	.12	.16*	.23**	.13	.01
Agreeableness	.29***	.08	.32***	−.04	.08	.00
Conscientiousness	.32***	.10	.09	.27***	.12	.02
Neuroticism	−.68***	.46	−.29***	−.48***	.51	.05
Openness	.08*	.01	.10	−.02	.01	.00
Competence	.33***	.11	.17*	.19**	.12	.01
Autonomy	.56***	.31	.39***	.20**	.31	.00
Relatedness	.48***	.23	.35***	.15*	.23	.00
Global health	.40***	.16	.07	.40***	.21	.04
Reduced functional ability	−.27***	.07	−.11	−.19**	.08	.01
Age	.26***	.07	.10	.19**	.07	.01
Gender (women)	−.05	.00	.05	−.11	.01	.01
Income	.20***	.04	.08	.15	.05	.00
	Analysis 3					
	Step 1: Depression		Step 2: Depression+LS			
Well-being correlates	β_Depression_	*R* ^2^	β_Depression_	β_LS_	*R* ^2^	Δ*R*^2^
Extraversion	.37***	.13	.19**	.21**	.14	.00
Agreeableness	.24***	.06	−.04	.32***	.09	.03
Conscientiousness	.35***	.12	.19**	.19**	.13	.00
Neuroticism	−.72***	.52	−.61***	−.13*	.50	−.01
Openness	.07	.01	.06	.00	.00	.00
Competence	.34***	.12	.08	.32***	.14	.03
Autonomy	.54***	.29	.06	.57***	.38	.09
Relatedness	.46***	.21	.02	.53***	.29	.08
Global health	.47***	.22	.14*	.39***	.25	.03
Reduced functional ability	−.29***	.08	−.11	−.22***	.09	.01
Age	.27***	.07	.31***	−.04	.08	.00
Gender (women)	−.07	.01	−.18**	.13	.02	.01
Income	.22***	.05	−.05	.32***	.08	.03
	Analysis 4					
	Step 1: Depression		Step 2: Depression+EWB			
Well-being correlates	β_Depression_	*R* ^2^	β_Depression_	β_EWB_	*R* ^2^	Δ*R*^2^
Extraversion	.37***	.13	.23**	.16*	.13	.00
Agreeableness	.24***	.06	−.04	.32***	.08	.03
Conscientiousness	.35***	.12	.27***	.09	.12	−.01
Neuroticism	−.72***	.52	−.48***	−.29***	.51	.00
Openness	.07	.01	−.02	.10	.01	.00
Competence	.34***	.12	.19**	.17*	.12	.00
Autonomy	.54***	.29	.20**	.39***	.31	.02
Relatedness	.46***	.21	.15*	.35***	.23	.02
Global health	.47***	.22	.40***	.07	.21	−.01
Reduced functional ability	−.29***	.08	−.19**	−.11	.08	.00
Age	.27***	.07	.19**	.10	.07	.00
Gender (women)	−.07	.01	−.11	.05	.01	.00
Income	.22***	.05	.15	.08	.05	.00

*Note*. Δ*R*^2^ = change in *R*^2^ from step 1 to step 2. β = standardized regression coefficient; CES-D 8 = 8-item Center for Epidemiologic Studies Depression Scale; EWB = emotional well-being; LS = life satisfaction.

* *p* < .05. ***p* < .01. ****p* < .001.
